# The effect of ankle tape on joint position sense after local muscle fatigue: a randomized controlled trial

**DOI:** 10.1186/s12891-017-1909-2

**Published:** 2018-01-09

**Authors:** Akram Jahjah, Dietmar Seidenspinner, Karl Schüttler, Antonio Klasan, Thomas J. Heyse, Dominik Malcherczyk, Bilal Farouk El-Zayat

**Affiliations:** Center of Orthopedics and Traumatology, University Hospital Marburg, Baldingerstrasse, 35033 Marburg, Germany

## Abstract

**Background:**

Ankle tape is widely used by athletes to prevent ankle sprain. Although there is growing evidence that ankle tape improve joint position sense, but yet it is not clear even if tape improve joint position sense after muscle fatigue, because fatigue impair joint position sense and raise the risk of ankle sprain. The aim of this study is to examine the effect of ankle tape on joint position sense after local muscle fatigue.

**Method:**

This trial is a randomized controlled trial. 34 healthy subjects participated in this trial. Subjects were randomized distributed into two groups: with tape and without tape. Active and passive absolute error and variable error mean values for two target positions of the ankle joint (15° inversions and inversion minus 5°) before and after fatigue protocol consisted of 30 consecutive maximal concentric/concentric contractions of the ankle evertors and invertors. In this trail joint position sense for all subjects was assessed using The Biodex System isokinetic dynamometer 3, this system is used also for fatigue protocol.

**Results:**

For the variable error (VE), significant mean effect was found for active joint position sense in 15° of inversion after muscle fatigue (*P* < 0, 05). It was a significant decrease in the work in the last third of inversion detected (*P* < 0, 05). There was no significant main effect found for fatigue index of eversion.

**Conclusion:**

Ankle tape can improve joint position sense at the fatigue session when joint position sense becomes worse. As a result, ankle tape may be useful to prevent ankle sprain during playing sports. We suggest athletes and individuals at risk of ankle sprain to apply taping before high-load activity.

**Trial registration:**

The study was retrospectively registered on the ISRCTN registry with study ID ISRCTN30042335 on 12th December 2017.

## Background

Ankle tape is commonly used by athletes to prevent ankle sprain in sports [1]. Epidemiological studies have established the ability of tape to minimize the number of acute ankle sprains during a playing season. The main role of ankle taping is to limit movement in normal range [2]. Some authors have investigated the protective role of taping through proprioception and awareness of ankle position [3–9].

Many risk factors have been reported in the literature for inversion ankle sprain, and are traditionally divided into intrinsic and extrinsic categories. Individual characteristic such as sex, weight, age, history of ankle sprain, joint laxity, functional instability, muscle strength, and proprioception are nominated as intrinsic factors. Outside effects such as quality of training, exercise load, play level, field conditions, shoe type, ankle bracing, and taping [10, 11].

Researchers have directly linked a fatigued state with impairment of joint position sense in either healthy or unhealthy individuals [12, 13]. It has been assumed that neuromuscular feedback can be inhibited through muscle fatigue around the joint [12, 14]. There is also growing evidence that fatigue influences the risk of inversion ankle sprains [15]. In support of fatigue as a risk factor, overall injury rates have been shown to increase towards the end of matches [16, 17]. In addition, an epidemiologic study found that most ankle sprains occurred in the last third of the first and second halves of matches [17].

We examined the effect of ankle tape on joint position sense after local muscle fatigue. This effect has not been clearly delineated in the literature. Several authors have demonstrated this effect, but only at rest and not under local muscle fatigue conditions. Secondarily, we examined the influence of tape itself on local muscle fatigue.

## Methods

### Subjects

Thirty-four healthy subjects participated in this randomized controlled trial. Subjects with a history of lower extremity injuries, neurologic deficits, rheumatologic disease, and hypermobility were excluded. None of the subjects had experience with using ankle tape. All subjects were asked to refrain from any heavy exercise 48 h prior to the test protocol. All subjects were informed about the study details and possible side effects; written informed consent was obtained from all subjects prior to study participation and the study protocol was approved by the ethical committee of Philipps-University Marburg (Germany). Baseline data for physical characteristics were collected for each subject and included age, sex, height, weight, and leg dominance (Table 1).

Subjects were randomly distributed into control and intervention groups by block randomization using Random Allocation Software (2004) [9], which was implemented by two independent investigators. In both groups, joint position sense was measured before and after local muscle fatigue. Modified methods from previous study protocols were is used in this trial [18].

### Measurement of the joint position sense

Joint position sense was assessed for all subjects using the Biodex System Isokinetic Dynamometer 3 and Biodex Advantage Software Package (Biodex Medical System Inc., Shirley, NY, USA). This instrument has been shown to be valid and reliable in prior publications [19]. Each subject was positioned barefoot in a beach chair position with the calf of the tested leg resting on the platform. Each foot was tightly secured to the dynamometer footplate with two straps to minimize movement between the sole of the foot and the footplate surface. The lower leg was also fixed to avoid knee movement. The tested extremity was in 45° flexion. The dominant foot of the subject was aligned with the axis of the dynamometer and the ankle joint was fixed at 15° of plantar flexion. Dynamometer and chair adjustments were made to align the midline of the knee. Active and passive joint position sense was tested in two positions: 15° of inversion and inversion minus 5°. All subjects were blindfolded throughout the examination. The complete test protocol was implemented with each subject before the main test in order to minimize the learning effect.

For passive testing, the subject’s foot was moved from the starting position minus 30° to one of two randomly predetermined target positions, and held for 5 s. Each subject was instructed to concentrate on the position of his or her foot. The foot was then passively returned to the starting position and started moving with a speed of 5°/s by the Biodex System. The subject was instructed to push on a stop button when he or she thought the test position had been reached.

For active testing, we repeated the same method. As soon as the foot was passively returned to the starting position, subjects were asked to reproduce actively the target position and to push on a stop button when he or she reached the target.

We chose mid-range motion in inversion and eversion; otherwise subjects were able to return their foot more easily to the target position.

The absolute error (AE) is the difference in absolute value in degrees between chosen and target position, and is a measure of the overall accuracy of positioning. The variable error (VE) is the difference in real values between chosen and target position, and is a measure of the variability of positioning.

Accuracy of joint position sense is inversely proportional with AE and VE scores. The test was repeated three times to calculate the mean score used for analysis.

### Fatigue protocol

Subjects were positioned on the chair with 30° of seatback tilt. The ankle was placed at 15° of plantar flexion. The knee of the tested ankle was placed at 45° flexion. The chair was adjusted to keep the knee between the hip and the foot in the same midline. To limit extreme movements during the test, two straps were fixed around the pelvis and proximal to the knee. Subjects performed 10 repetitions of consecutive maximal concentric/concentric contractions of the ankle in order to become familiarized with the device and to minimize the restricted effect of the tape. They rested their foot for 10 min to recover muscle strength.

The local load applied to the ankle joint consisted of 30 consecutive maximal concentric/concentric contractions of the ankle evertors and invertors in the range of motion ROM (30° of eversion and 30° of inversion) at an angular velocity of 120°/s. Subjects were verbally encouraged and motivated to work hard and perform at their maximum potential. Immediately after the fatigue protocol, joint position sense testing was initiated using the same methods as in the testing before fatigue, to minimize the effects related to recovery from fatigue.

Local fatigue was evaluated by measuring changes in fatigue index (%), work (W) in Joules (J), work in the first third (W1), and work in the last third (W3). A fatigue index was adopted from Rebeiro et al. [20] and was calculated according following formula:

$$ \mathrm{Fatigue}\  \mathrm{index}=\left(\mathrm{initial}\  \mathrm{peak}\  \mathrm{torque}\hbox{-} \mathrm{final}\  \mathrm{peak}\  \mathrm{torque}\right)/\mathrm{initial}\  \mathrm{peak}\  \mathrm{torque}\ast 100. $$

Initial peak torque and final peak torque were calculated as the average of the first and the last five repetition peak torques of the fatigue protocol. Thus, an increased fatigue index value represents an increased level of fatigue.

### Intervention

Preventive taping for injuries to the lateral aspect of the ankle joint was used and applied according to Macdonald’s method [21]. This works by supporting the lateral aspect of the ankle by mechanical support supplied by the tape and its interface with the anchors, and proprioceptive response triggered by the pull of the skin when moving the foot during activity. The material used for ankle taping was Leukotape® classic (3.75 cm, BSN medical GmbH, Hamburg, Germany) and NOBA®-PRETAPE (10 cm, NOBAMED Paul Danz AG, Wetter/Ruhr, Germany).

### Statistical results

Data analysis was performed using SPSS statistical software (version 17.0, SPSS Inc., Chicago, IL, USA). Using the primary question of the effect of the ankle tape on joint position sense before and after fatigue, active and passive joint position senses for two target positions of the ankle joint (15° inversion and inversion minus 5°) were analysed individually using the paired t test to evaluate the effects of fatigue on joint position sense with and without ankle tape. The independent t test was used to compare differences between absolute error and variable error for the mean values of the two target positions. The secondary question was to determine the effects of ankle tape on fatigue. Therefore, the variables of fatigue index (FI), work (W), work at the first third (W1), and work at the last third (W3) were analysed in two directions, eversion and inversion, with and without ankle tape. The independent t test was used to compare the differences among FI, W, W1, and W3 mean values for eversion and inversion between groups. A *p*-value of less than 0.05 was considered statistically significant.

## Results

### Descriptive data

Data analysis was performed using SPSS statistical software (version 17.0, SPSS Inc., Chicago, IL, USA). Using the primary question of the effect of the ankle tape on joint position sense before and after fatigue, active and passive joint position senses for two target positions of the ankle joint (15° inversion and inversion minus 5°) were analysed individually using the paired t test to evaluate the effects of fatigue on joint position sense with and without ankle tape. The independent t test was used to compare differences between absolute error and variable error for the mean values of the two target positions. The secondary question was to determine the effects of ankle tape on fatigue. Therefore, the variables of fatigue index (FI), work (W), work at the first third (W1), and work at the last third (W3) were analysed in two directions, eversion and inversion, with and without ankle tape. The independent t test was used to compare the differences among FI, W, W1, and W3 mean values for eversion and inversion between groups. A *p*-value of less than 0.05 was considered statistically significant.

**Table 1:** Subject characteristic in mean ± SD

Variables	Control Group (n=17)	Intervention Group (n=17)	All Subjects (n=34)
Age (year)	26,8 ± 2,6	26,7 ± 3,7	26,8 ± 3,2
Height (cm)	173,1 ± 9,7	175,2 ± 11,1	174,1 ± 10,3
Weight (kg)	68,7 ± 17,2	74,2 ± 15,5	71,5 ± 16,4

### Joint position sense

**Fig. 1:**
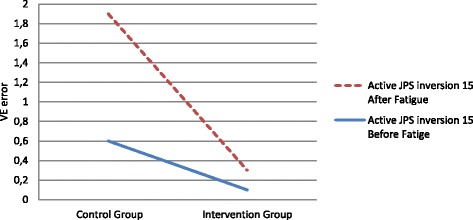
Mean variable error (VE) for 15° of inversion before and after muscle fatigue

**Fig. 2:**
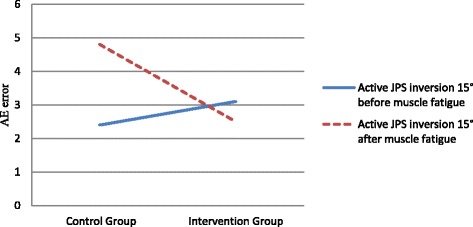
Mean absolute error (AE) for 15° of inversion before and after muscle fatigue

For the variable error (VE), a significant mean effect was found for active joint position sense in 15° of inversion after muscle fatigue (*p* = 0.034, Table 2, Fig. 1). The subject’s ability to perceive joint position sense for 15° of inversion with ankle tape after muscle fatigue significantly improved; the mean error was 1.3° ± 5.7° in the group without tape compared with the mean error of 0.2° ± 3.4° in the group with tape. The mean absolute error (AE) before muscle fatigue for an active joint position sense in 15° of inversion was 2.4° ± 2.7° in the control group, versus 3.1° ± 2.1° in the intervention group (*p* = 0.699). After muscle fatigue, the mean error for the same position in the intervention group was 2.5° ± 2.3° versus 4.8° ± 3.1° in the control group. However, this difference was not found to be statistically significant (*p* = 0.446, Table 3, Fig. 2). No difference was found in absolute error for active joint position sense inversion minus 5° before fatigue between the control and intervention groups (2.4° ± 1.4° vs. 2.3° ± 1.3°, *p* = 0.62); likewise, no difference was found between groups after fatigue (3.1° ± 2.4° vs. 1.9° ± 1.6°, *p* = 0.319).

**Table 2:** Effect of ankle tape on joint position sense variable error (VE) in degrees mean ± SD

	Variables	Control Group	Intervention Group	*P* Value
Before Fatigue	Active JPS inversion minus 5°	2,2 ± 1,6	2,2 ± 1,4	0,513
	Active JPS inversion 15°	0,6 ± 3,6	0,1 ± 3,8	0,562
	Passive JPS inversion minus 5°	2,4 ± 1,7	3,5 ± 1,8	0,686
	passive JPS inversion 15°	0,02 ± 2,3	−2 ± 2,5	0,381
After Fatigue	Active JPS inversion minus 5°	1,6 ± 2,04	2,4 ± 3,2	0,243
	Active JPS inversion 15°	1,3 ± 5,7	0,2 ± 3,4	0,034*
	Passive JPS inversion minus 5°	3,2 ± 2,5	3,4 ± 1,6	0,263
	passive JPS inversion 15°	−0,8 ± 4,2	−2,3 ± 3,4	0,379

^*^Significant

**Table 3:** Effect of ankle tape on joint position sense absolute error (AE) in degrees mean ± SD

	Variables	Control Group	Intervention Group	*P* Value
Before Fatigue	Active JPS inversion minus 5°	2,4 ± 1,4	2,3 ± 1,3	0,62
	Active JPS inversion 15°	2,4 ± 2,7	3,1 ± 2,1	0,699
	Passive JPS inversion minus 5°	2,4 ± 1,7	3,6 ± 1,7	0,558
	Passive JPS inversion 15°	1,5 ± 1,7	2,6 ± 1,8	0,542
After Fatigue	Active JPS inversion minus 5°	1,9 ± 1,6	3,1 ± 2,4	0,319
	Active JPS inversion 15°	4,8 ± 3,1	2,5 ± 2,3	0,446
	Passive JPS inversion minus 5°	3,3 ± 2,4	3,4 ± 1,6	0,297
	Passive JPS inversion 15°	3,3 ± 2,6	3,7 ± 1,7	0,287

**Table 4:** Effect of ankle tape on muscle fatigue ± SD

	Variables	Control Group	Intervention Group	*P* Value
Eversion	Fatigue Index	19,6 ± 18,2	18,1 ± 39,5	0,363
	Work	307,3 ± 98,7	327,5 ± 101,8	0,768
	Work 1_ Third	112,4 ± 37,9	120,3 ± 42,9	0,653
	Work3_Third	87,4 ± 30,7	93 ± 28,6	0,756
Inversion	Fatigue Index	11,3 ± 23,4	24,6 ± 16,4	0,131
	Work	319,1 ± 88,6	327,3 ± 126,6	0,166
	Work 1_ Third	113,9 ± 49,4	122,9 ± 49,4	0,252
	Work3_Third	94,4 ± 23,4	87,2 ± 40,3	0,019*

^*^Significant

### Muscle fatigue

**Fig. 3:**
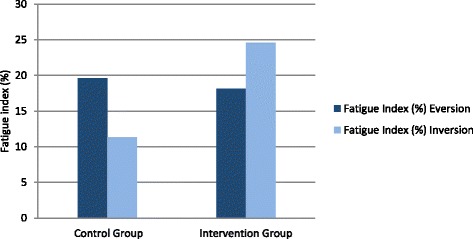
Fatigue index (%) of eversion and inversion in control and intervention group

**Fig. 4:**
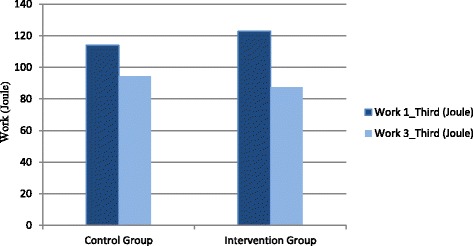
Work in the first and the last third of muscle fatigue protocol, the difference of work at the last session between control and intervention group was significant (*P* < 0.05)

There was no significant effect found for the fatigue index of eversion (19.6 ± 18.2 vs. 18.1 ± 39.5, *p* = 0.363) in the control vs. intervention groups (Table 4, Fig. 3). In inversion, there was no significant effect found for fatigue index, although the difference appears large (24.6 ± 16.4 vs. 11.3 ± 23.4, *p* = 0.131, Fig. 3). A significant decrease in work in the last third of inversion was detected (*p* = 0.019, Table 4, Fig. 4), showing that muscles fatigued faster in the group with tape than in the group without tape.

## Discussion

To our knowledge, this study is the first to describe the effect of ankle tape on joint position sense after local muscle fatigue, and the first to describe the effect of ankle tape on muscle fatigue. The combination of these two issues may provide a better understanding of the influence that tape produces. We demonstrated that ankle tape improves joint position sense at an active 15° of inversion after muscle fatigue.

An increase in fatigue index in the intervention group (24.6% ± 16.4) was observed compared to the control group (11.3% ± 23.4) in inversion. This result can be interpreted as evidence that taping provides support and limits functional motion, especially for inversion (Fig. 3). We also found a significant effect of ankle tape on the work in the last third of the fatigue protocol (*p* = 0.019) in inversion, but no significant effect in eversion (*p* = 0.756) (Table 4). Others reported that the use of tape limited inversion and maximized plantar flexion [22]. In this interpretation, tape affects the invertor muscle group, which was more fatigued in the intervention versus control group.

Evertor muscles have been shown to play an important role in preventing inversion ankle sprains [18] because of eccentric contractions that evert the ankle when it comes into inversion, before tissue strain becomes excessive [23]. This hypothesis seems to be acceptable because of the relationship between peroneal muscle weakness and chronically unstable ankles [24, 25]. Most authors confirmed the role of evertor muscles in preventing ankle sprain [18], finding no relationship between invertor muscle strength and ankle sprains. These findings, in combination with presented results indicate that the fatigue of invertor muscles have no direct influence on ankle sprain.

The results of this trial are comparable to that of Robbins et al. [3] and Miralles et al. [22]; however, these two studies investigated the effect of tape on joint position sense in ankle plantar flexion and dorsiflexion, while this trial tested joint position sense in ankle inversion and eversion. In 1995, Robbins et al. were the first to study the effect of ankle tape on foot position awareness before and after exercise. They significant improvement of position sense in absolute error in ankles taped before and after exercise, specifically in an exercise protocol that consisted of 30 min of running and playing basketball. They compared two groups wearing tape and athletic footwear. Their results indicate that taping influenced foot position awareness mainly when the surface slope was greater than 10° plantar flexion, which is probably most relevant to ankle sprains. These results reinforce our findings that the effect of tape is greater when there is a larger range of motion; in this trial, there was a greater effect in 15° of inversion compared to inversion minus 5°. This may be more relevant to ankle sprain, because lateral ankle sprain occurs most frequently when there is a large range of inversion.

Miralles et al. studied the effect of tape on joint position sense, before the tape was placed compared with placement after 48 h. The intervention group showed a trend towards improvement after 48 h for all positions compared to the control group. This improvement was significant in dorsiflexion and in the neutral position. In contrast to Robbins’s findings, that proprioception was present mainly in ranges of movement over 10°; Miralles’s results indicate that joint position sense may improve even for positions under 10°. The results of this study reinforce Robbins’s, as this effect is reported in 15° of inversion.

Muscle fatigue protocols can be divided into three main categories: muscle fatigue over time [26], fatigue due to the number of repetitions [20], and fatigue due to the intensity of peak torque [15]. In this trial, muscle fatigue was based on the number of repetitions of eversion and inversion at the ankle joint. Through isometric contraction, subjects do not move the foot; it stays in the neutral position. Thus, it is not possible to notice the effect of tape on muscle fatigue. Also, if all subjects have a maximum voluntary contraction MVC equal to 50%, it would not be possible to compare muscle fatigue results since all subjects would have the same level of fatigue.

These results showed an alteration of joint position sense in AE in the control group after muscle fatigue. This was in an active 15° of inversion before (2.4° ± 2.7°) and after fatigue (4.8° ± 3.1°) (Table 3). These results support the findings of other researchers, such as Mohammadi et al. [15], who investigated the effect of fatigue due to contraction of evertor muscles on joint position sense in male soccer players. They reported that joint position sense in AE in active 15° of inversion was 1.9° ± 1.2° before fatigue and 3.2° ± 1.9° after fatigue. They also reported this difference in inversion minus 5°, which was 1.7° ± 0.7° before fatigue and 2.9° ± 1.3° after fatigue; this is in contrast to our presented results in this position. Forestier et al. [12] investigated how an isometric exhaustion test affects position sense at the ankle using a matching task. In their trial, joint position sense in dorsiflexion and plantar flexion were measured. The results showed an alteration of joint position sense after fatigue: in AE it was 2.2° ± 1.3° before fatigue and 3.2° ± 1.8° after fatigue. These results also support the findings of this trial.

Several mechanisms are involved in how fatigue may influence joint position sense. It has been suggested that increased joint laxity may play a role in joint position sense changes after fatigue, because fatigue increases laxity of ankle joint ligaments [27]. A further possible mechanism may be the effect of fatigue on peripheral receptors. The relative contribution of joint and muscle receptors to measure joint position sense deficits after a fatigue protocol also remains controversial. It is generally accepted that the greatest contribution to position sense is from muscular receptors [28, 29]. Several reports have claimed that fatigue presumably affects muscle receptors more than joint receptors; this means that a decrease in joint position sense may be due to loss of muscle receptor input [30–32].

These two mechanisms are important for the interpretation of mentioned results, which demonstrate that tape improves joint position sense after local fatigue and these mechanisms actually respond to the mechanisms suggestive of the effect of fatigue on joint position sense. The first mechanism is the mechanical role of tape in limiting the range of motion, which can minimize joint laxity after muscle fatigue. This effect can improve joint position sense [4]. The second mechanism is to improve muscles receptors, which are impaired after muscle fatigue and can thus improve joint position sense after fatigue [33].

### Limitations

Taping by all subjects is not 100% reliable because it has been applied by humans, and it is related to the type of foot, skin, and tape. Further, the foot was tightly secured to the dynamometer footplate with two straps; the straps might have affected the results because they play the role of another external support to the foot.

### Recommendations

More research is required to investigate the effect of tape on other groups of professional athletes, such as basketball or football players. Limiting the sample population to males or females may also be beneficial due to sex differences in laxity and muscle strength. Reliability and validity of this fatigue protocol need further investigation, by assessing MVC before and after taping. Epidemiological studies are needed to define the preventive role of tape in injuries of the ankle joint.

## Conclusions

In conclusion, we demonstrated that joint position sense significantly improves after taping of the ankle joint in 15° of inversion after local muscle fatigue. In addition, we proved that tape fatigues the ankle in the direction of inversion. These results show that ankle tape can improve joint position sense at the fatigue session, when joint position sense becomes less acute. Thus, ankle tape may be useful before high intensity-demanding functional activity.

## Abbreviations

AE: Absolute error
FI: Fatigue Index
MVC: Maximum voluntary contraction
ROM: Range of motion
VE: Variable error
W: Work
